# Supplementary material for: Efficient pheromone navigation via antagonistic detectors in Caenorhabditis elegans male
Source: Nat Commun. 2026 Feb 13;17:2738. doi: 10.1038/s41467-026-69392-2 (PMC13013886; doi:10.1038/s41467-026-69392-2)
Supplement: Supplementary file 2 — Description of Additional Supplementary Files [file 41467_2026_69392_MOESM2_ESM.pdf]

Description of Additional Supplementary Files

Supplementary Movie 1:
Simulated navigation of worms without head input. The transparent yellow sphere indicates the pheromone source. Worms are small spheres colored by their instantaneous confidence $Q^H - Q^T$. A typical failed trajectory is shown in yellow. Left window: with tail inputs. Right window: without tail inputs.

Supplementary Movie 2:
Simulated navigation of worms with head input. The transparent yellow sphere indicates the pheromone source. Worms are small spheres colored by their instantaneous confidence $Q^H - Q^T$. A typical successful trajectory is shown in yellow.
Left window: with tail inputs. Right window: without tail inputs.
