# Supplementary material for: Efficient pheromone navigation via antagonistic detectors in Caenorhabditis elegans male
Source: Nat Commun. 2026 Feb 13;17:2738. doi: 10.1038/s41467-026-69392-2 (PMC13013886; doi:10.1038/s41467-026-69392-2)
Supplement: Supplementary file 5 — Reporting Summary [file 41467_2026_69392_MOESM5_ESM.pdf]

nature portfolio

Corresponding author(s): Paul W. Sternberg
Last updated by author(s): 1/1/2026

Reporting Summary

Nature Portfolio wishes to improve the reproducibility of the work that we publish. This form provides structure for consistency and transparency in reporting. For further information on Nature Portfolio policies, see our Editorial Policies and the Editorial Policy Checklist.

Statistics

For all statistical analyses, confirm that the following items are present in the figure legend, table legend, main text, or Methods section.

n/a	Confirmed	
☐	☒	The exact sample size (*n*) for each experimental group/condition, given as a discrete number and unit of measurement
☐	☒	A statement on whether measurements were taken from distinct samples or whether the same sample was measured repeatedly
☐	☒	The statistical test(s) used AND whether they are one- or two-sided<br>*Only common tests should be described solely by name; describe more complex techniques in the Methods section.*
☐	☒	A description of all covariates tested
☐	☒	A description of any assumptions or corrections, such as tests of normality and adjustment for multiple comparisons
☐	☒	A full description of the statistical parameters including central tendency (e.g. means) or other basic estimates (e.g. regression coefficient) AND variation (e.g. standard deviation) or associated estimates of uncertainty (e.g. confidence intervals)
☐	☒	For null hypothesis testing, the test statistic (e.g. *F*, *t*, *r*) with confidence intervals, effect sizes, degrees of freedom and *P* value noted<br>*Give P values as exact values whenever suitable.*
☒	☐	For Bayesian analysis, information on the choice of priors and Markov chain Monte Carlo settings
☐	☒	For hierarchical and complex designs, identification of the appropriate level for tests and full reporting of outcomes
☐	☒	Estimates of effect sizes (e.g. Cohen's *d*, Pearson's *r*), indicating how they were calculated

Our web collection on statistics for biologists contains articles on many of the points above.

Software and code

Policy information about availability of computer code

Data collection	WormLab Tracking system (MBF Biosciences) used for data collection.
Data analysis	Software and algorithms<br>Fiji (ImageJ distribution) Version 2.16.0 (Oct 15, 2024) Schindelin et al., 2012 https://imagej.net/software/fiji/<br><br>MaMuT Version 0.27.0 Wolff et al., 2018 https://imagej.net/plugins/mamut/<br><br>Targettrack Version: GitHub rahi-lab/targettrack, Park CF et al., Nat Methods 21:142–149 (2024) https://www.nature.com/articles/s41592-023-02096-3<br><br>ilastik Version 1.4.0.post1 Berg et al., 2019 https://www.ilastik.org<br><br>WormLab Tracking System (MBF Bioscience) Version 2024.1.1 MBF Bioscience (WormLab Release Notes) https://www.mbfbioscience.com/products/wormlab/<br><br>Custom code: pheromone-traj-analysis Version: GitHub edmondztt/pheromone-traj-analysis, This study https://github.com/edmondztt/pheromone-traj-analysis

For manuscripts utilizing custom algorithms or software that are central to the research but not yet described in published literature, software must be made available to editors and reviewers. We strongly encourage code deposition in a community repository (e.g. GitHub). See the Nature Portfolio guidelines for submitting code & software for further information.

Data

Policy information about availability of data

All manuscripts must include a data availability statement. This statement should provide the following information, where applicable:

- Accession codes, unique identifiers, or web links for publicly available datasets
- A description of any restrictions on data availability
- For clinical datasets or third party data, please ensure that the statement adheres to our policy

Source data are provided with this paper.

Research involving human participants, their data, or biological material

Policy information about studies with human participants or human data. See also policy information about sex, gender (identity/presentation), and sexual orientation and race, ethnicity and racism.

Reporting on sex and gender	This study involves the invertebrate C. elegans; no human participants or vertebrate animals were included. Sex/gender guidance for human/vertebrate research is not applicable; where relevant, we specify worm sex (hermaphrodite vs. male) in the Methods and figure legends.
Reporting on race, ethnicity, or other socially relevant groupings	n/a
Population characteristics	n/a
Recruitment	n/a
Ethics oversight	n/a

Note that full information on the approval of the study protocol must also be provided in the manuscript.

Field-specific reporting

Please select the one below that is the best fit for your research. If you are not sure, read the appropriate sections before making your selection.

☒ Life sciences ☐ Behavioural & social sciences ☐ Ecological, evolutionary & environmental sciences

For a reference copy of the document with all sections, see nature.com/documents/nr-reporting-summary-flat.pdf

Life sciences study design

All studies must disclose on these points even when the disclosure is negative.

Sample size	Sample sizes were determined from pilot effect sizes and prior literature; the final n per group is reported in the figure legends.
Data exclusions	In behavioral analysis: Exclusion criteria: (1) any worm that physically contacted another worm during the assay; (2) any worm that reached the plate edge.
Replication	All key results were confirmed in at least 3 independent biological replicates (separate cohorts/plates on different days), with 3-7 technical replicates where applicable.
Randomization	Animals/plates were randomly assigned to conditions.
Blinding	Data collection and analysis were performed blind to genotype/treatment, with unblinding only after primary analyses were finalized.

Reporting for specific materials, systems and methods

We require information from authors about some types of materials, experimental systems and methods used in many studies. Here, indicate whether each material, system or method listed is relevant to your study. If you are not sure if a list item applies to your research, read the appropriate section before selecting a response.

Materials & experimental systems

n/a	Involved in the study
[x]	[ ] Antibodies
[x]	[ ] Eukaryotic cell lines
[x]	[ ] Palaeontology and archaeology
[ ]	[x] Animals and other organisms
[x]	[ ] Clinical data
[x]	[ ] Dual use research of concern
[x]	[ ] Plants

Methods

n/a	Involved in the study
[x]	[ ] ChIP-seq
[x]	[ ] Flow cytometry
[x]	[ ] MRI-based neuroimaging

Animals and other research organisms

Policy information about studies involving animals; ARRIVE guidelines recommended for reporting animal research, and Sex and Gender in Research

Laboratory animals

Caenorhabditis elegans
Experimental models: Organisms/strains (C. elegans):him-5(e1490) V (CGC, CB4088), male-enriched mutant; srd-1(eh1) (CGC, CB5414), srd-1 mutant; syEx1972 [srd-1P::mCherry]; syEx1974 [unc-119::gfp] (This study, PS10451), srd-1 reporter; pan-neural marker; syEx1973 [rab-3P::mCherry]; syIs912 [srd-1P::Gal4(sk)::VP64]; syIs300 [15xUAS::GFP] (This study, PS10448), srd-1 driver; GFP effector; pan-neural marker; syEx1972 [srd-1P::mCherry]; syIs913 [pkd-2P::Gal4(sk)::VP64]; syIs300 [15xUAS::GFP] (This study, PS10449), srd-1 reporter; pkd-2 driver; GFP effector; syIs912 [srd-1P::Gal4(sk)::VP64] (This study, PS9477), srd-1 driver; syIs914 [srd-1P::Gal4(sk)::VP64] (This study, PS9478), srd-1 driver; syIs912 [srd-1P::Gal4(sk)::VP64]; syIs300 [15xUAS::GFP] (This study, PS9473), srd-1 driver; GFP effector; syIs913 [pkd-2P::Gal4(sk)::VP64]; syIs300 [15xUAS::GFP] (This study, PS9681), pkd-2 driver; GFP effector; syIs838 [lin-48dP::NLS::cGAL(DBD)::gp41-1::N-intein::let-858 3'UTR, srd-1P::NLS::gp41-1::C-intein::cGAL(AD)::let-858 3'UTR] (This study, PS10007), PHD driver; syIs838 [lin-48dP::NLS::cGAL(DBD)::gp41-1::N-intein::let-858 3'UTR, srd-1P::NLS::gp41-1::C-intein::cGAL(AD)::let-858 3'UTR]; syIs300 [15xUAS::GFP] (This study, PS9573), PHD driver; GFP effector; syIs888 [gpa-14P::NLS::cGAL (DBD)::gp41-1::N-intein::let-858 3'UTR, rig-3P::NLS::gp41-1::C-intein::cGAL(AD)::let-858 3'UTR] (This study, PS10179), AVA driver; syIs888 [gpa-14P::NLS::cGAL(DBD)::gp41-1::N-intein::let-858 3'UTR, rig-3P::NLS::gp41-1::C-intein::cGAL(AD)::let-858 3'UTR]; syIs300 [15xUAS::GFP] (This study, PS10087), AVA driver; GFP effector; syIs564 [odr-10P::Gal4(sk)::VP64]; syIs300 [15xUAS::GFP] (This study, PS8293), AWA driver; GFP effector; syIs340 [15xUAS::hChR(H134R)::EYFP::let-858 3'UTR]; him-5(e1490) V (This study, PS9782), Chrimson effector; him-5 background; syIs340 [15xUAS::hChR(H134R)::EYFP::let-858 3'UTR] (This study, PS7043), Chrimson effector; syIs371 [15xUAS::HisCL::SL2::GFP::let-858 3'UTR] (This study, PS7199), HisCl effector; syIs612 [15xUAS::GCaMP7b::SL2::mKate2] (This study, PS9046), GCaMP7b effector; syIs912 [srd-1P::Gal4(sk)::VP64]; syIs612 [15xUAS::GCaMP7b::SL2::mKate2] (This study, PS9680), srd-1 driver; GCaMP7b effector; syIs912 [srd-1P::Gal4(sk)::VP64]; syIs371 [15xUAS::HisCL::SL2::GFP::let-858 3'UTR] (This study, PS10005), srd-1 driver; HisCl effector; syIs912 [srd-1P::Gal4(sk)::VP64]; syIs340 [15xUAS::hChR(H134R)::EYFP::let-858 3'UTR]; him-5 (e1490) V (This study, PS9990), srd-1 driver; Chrimson effector; him-5 background; syIs838 [lin-48dP::NLS::cGAL(DBD)::gp41-1::N-intein::let-858 3'UTR, srd-1P::NLS::gp41-1::C-intein::cGAL(AD)::let-858 3'UTR]; syIs371 [15xUAS::HisCL::SL2::GFP::let-858 3'UTR] (This study, PS10189), PHD driver; HisCl effector; syIs838 [lin-48dP::NLS::cGAL(DBD)::gp41-1::N-intein::let-858 3'UTR, srd-1P::NLS::gp41-1::C-intein::cGAL(AD)::let-858 3'UTR]; syIs340 [15xUAS::hChR(H134R)::EYFP::let-858 3'UTR]; him-5(e1490) V (This study, PS10187), PHD driver; Chrimson effector; him-5 background; syIs888 [gpa-14P::NLS::cGAL(DBD)::gp41-1::N-intein::let-858 3'UTR, rig-3P::NLS::gp41-1::C-intein::cGAL(AD)::let-858 3'UTR]; syIs612 [15xUAS::GCaMP7b::SL2::mKate2] (This study, PS10181), AVA driver; GCaMP7b effector; syIs564 [odr-10P::Gal4(sk)::VP64]; syIs340 [15xUAS::hChR(H134R)::EYFP::let-858 3'UTR] (This study, PS9387), AWA driver; Chrimson effector; hpIs675 [rgef-1P::GCaMP6s::3xNLS::mNeptune + lin-15(+)]; lin-15(n765) X; him-5(e1490) V (This study, ZM9627), pan-neuronal nuclear mNeptune and GCaMP6s; hpIs675 [rgef-1P::GCaMP6s::3xNLS::mNeptune + lin-15(+)]; lin-15(n765) X; him-5(e1490) V; srd-1(eh1) (This study, PS10450), ZM9627 in srd-1(eh1) background; lite-1(ce314); JAC66 [ift-20P::BFP; eat-4P::cyOFP1; unc-17P::mScarlet; acr-5P::BFP]; hpIs675 [rgef-1P::GCaMP6s::3xNLS::mNeptune + lin-15(+)]; lin-15 (n765) X (This study, ADS1046), pan-neuronal nuclear mNeptune/GCaMP6s plus neuron-ID markers; lite-1(ce314); JAC66 [ift-20P::BFP; eat-4P::cyOFP1; unc-17P::mScarlet; acr-5P::BFP]; hpIs675 [rgef-1P::GCaMP6s::3xNLS::mNeptune + lin-15(+)]; lin-15 (n765) X; syIs912 [srd-1P::Gal4(sk)::VP64]; syIs340 [15xUAS::hChR(H134R)::EYFP::let-858 3'UTR]; him-5(e1490) V (This study, PS10153), ADS1046 with srd-1 driver and Chrimson; him-5 background; Kp1368 [myo-2P::NLS::mCherry]; syIs371 [15xUAS::HisCL::SL2::GFP::let-858 3'UTR] (This study, PS8720), muscle driver; HisCl effector; syIs334 [rab-3P::GAL4::gp41::N::let-858 3'UTR]; syIs340 [15xUAS::hChR(H134R)::EYFP::let-858 3'UTR]; him-5(e1490) V (This study, PS8026), pan-neural driver; Chrimson effector; him-5 background.

E. coli OP50 Caenorhabditis Genetics Center (CGC) WormBase: OP50; WormBase: WBStrain00041969 Food source for all C. elegans experiments (unless otherwise stated)

Wild animals

n/a

Reporting on sex

This study involves the invertebrate C. elegans; no human participants or vertebrate animals were included. Sex/gender guidance for human/vertebrate research is not applicable; where relevant, we specify worm sex (hermaphrodite vs. male) in the Methods and figure legends. Worm sex is identified by morphology.

Field-collected samples

n/a

Ethics oversight

n/a

Note that full information on the approval of the study protocol must also be provided in the manuscript.

Plants

Seed stocks	n/a
Novel plant genotypes	n/a
Authentication	n/a
